# Pathogenic mechanisms and therapeutic implications of extracellular matrix remodelling in cerebral vasospasm

**DOI:** 10.1186/s12987-023-00483-8

**Published:** 2023-11-04

**Authors:** Ziliang Hu, Xinpeng Deng, Shengjun Zhou, Chenhui Zhou, Menglu Shen, Xiang Gao, Yi Huang

**Affiliations:** 1 Department of Neurosurgery, The First Affiliated Hospital of Ningbo University, Liuting Street 59, Ningbo, 315010 Zhejiang China; 2 Cixi Biomedical Research Institute, Wenzhou Medical University, Cixi, 315302 Zhejiang China; 3 Cixi Third People’s Hospital, Cixi, 315324 Zhejiang China; 4 Key Laboratory of Precision Medicine for Atherosclerotic Diseases of Zhejiang Province, Ningbo, 315010 Zhejiang China

**Keywords:** Extracellular matrix, Subarachnoid hemorrhage, Cerebral vasospasm, Vascular remodelling, Homeostasis

## Abstract

Cerebral vasospasm significantly contributes to poor prognosis and mortality in patients with aneurysmal subarachnoid hemorrhage. Current research indicates that the pathological and physiological mechanisms of cerebral vasospasm may be attributed to the exposure of blood vessels to toxic substances, such as oxyhaemoglobin and inflammation factors. These factors disrupt cerebral vascular homeostasis. Vascular homeostasis is maintained by the extracellular matrix (ECM) and related cell surface receptors, such as integrins, characterised by collagen deposition, collagen crosslinking, and elastin degradation within the vascular ECM. It involves interactions between the ECM and smooth muscle cells as well as endothelial cells. Its biological activities are particularly crucial in the context of cerebral vasospasm. Therefore, regulating ECM homeostasis may represent a novel therapeutic target for cerebral vasospasm. This review explores the potential pathogenic mechanisms of cerebral vasospasm and the impacts of ECM protein metabolism on the vascular wall during ECM remodelling. Additionally, we underscore the significance of an ECM protein imbalance, which can lead to increased ECM stiffness and activation of the YAP pathway, resulting in vascular remodelling. Lastly, we discuss future research directions.

## Highlights

1. Changes in extracellular matrix homeostasis in response to stress after subarachnoid hemorrhage.
2. Role of extracellular matrix in the pathogenesis of cerebral vasospasm.
3. The imbalance of extracellular matrix homeostasis is an important cause of vascular remodelling.
4. YAP pathway regulates the proliferation of vascular cells through the extracellular matrix.

## Background

Aneurysmal subarachnoid hemorrhage (SAH) is a profoundly devastating acute-onset ailment with a mortality rate of up to 50% [1]. While advancements in medical technology have enhanced survival rates by 17% compared to decades past [2], approximately 15% of patients endure severe disabilities, and merely 20–35% of patients ultimately experience recovery [3]. One of the complications stemming from SAH is cerebral vasospasm (CV), which causes delayed cerebral ischaemia, the principal factor contributing to adverse outcomes in SAH [4]. Delayed CV, which refers to the sustained contraction of cerebral blood vessels within 72 h of SAH, manifests with an incidence rate of 70%. CV can induce ischaemia and hypoxia in critical brain regions, resulting in severe neurological damage [5]. Therefore, comprehending the potential pathogenesis of CV is crucial for its prevention and the enhancement of patient prognoses. Many candidate drugs for CV prevention exist (Table 1), but only one drug, namely nimodipine, is currently approved for CV treatment [6]. For an extended period, nimodipine has been acknowledged for its ability to significantly ameliorate severe neurological deficits arising from vasospasm following SAH and its capacity to enhance both mortality and neurological function. However, recent studies have revealed that nimodipine cannot reverse vasospasm [7, 8]. In SAH, the onset of vasospasm may be attributed to the excessive contraction of vascular smooth muscle cells (VSMCs) triggered by a substantial influx of blood, thereby leading to luminal vessel narrowing. This process is reversible; however, during this phase, the vascular wall structure may undergo alterations due to the proliferation and remodelling of VSMCs, culminating in vessel-wall thickening and stiffening. This may explain the prolonged vasoconstriction observed in SAH and the inefficiency of vasodilator treatments [9, 10].

**Table 1:** Research status of currently available candidate drugs for the prevention of cerebral vasospasm

Drug	Category	Effect	Therapeutic effect
Nimodipine	L-type Ca2+ channel blockers	It inhibits cortical diffuse ischemia, enhances endogenous fibrinolytic activity, reduces the incidence of microthrombus, and improves microcirculation [6]	While there is currently no compelling evidence supporting nimodipine’s preventive effect on large artery spasms or any angiographic evidence of cerebral vasodilation [120], its significant improvement of neurological function outcomes and reduction in mortality rates following subarachnoid hemorrhage are well-established [121]. Thus, nimodipine is the preferred medication for preventing adverse prognostic outcomes resulting from DCI subsequent to aneurysmal subarachnoid hemorrhage [6]
Nifedipine	L-type Ca2+ channel blockers	It exhibits regional selectivity for cerebral vascular smooth muscle [6]	It exerts a favourable effect on delayed cerebral ischemia and vascular spasm, but it does not improve mortality or disability rates. Furthermore, its efficacy is inferior compared to nimodipine [121]
Fosinopril	Rho-associated protein kinase inhibitors	By increasing MLCP activity to reduce the sensitivity of vascular smooth muscle and pericytes to Ca2+, it can dilate blood vessels. Furthermore, it can inhibit the generation of free radicals in white blood cells, impede the migration of white blood cells, reduce blood viscosity, and enhance eNOS activity [6]	It exerts a favourable effect on delayed cerebral ischemia and vascular spasm, but it does not improve mortality or disability rates. In Japan, it is exclusively employed as a medication for the prevention of vasospasm [121]
Magnesium sulphate	Voltage-gated Ca2+ channel blockers	It induces vasodilation by blocking voltage-dependent Ca2 + channels [8]	It exerts a favourable effect on delayed cerebral ischemia and vasospasm; however, it has not demonstrated improvements in mortality or disability rates. Notably, it is not a conventional medication [121]
Xylometazoline	Phosphodiesterase type 3 inhibitors (PDE3 inhibitors)	By inhibiting PDE3 and increasing the concentration of cyclic adenosine monophosphate (cAMP) to activate protein kinase A, it inactivates Ca2+-dependent myosin light chain kinase. Furthermore, it elevates NO levels in endothelial cells, thereby relaxing blood vessels [6, 122]	Xylometazoline has been shown to improve neurological outcomes and reduce mortality rates in patients with aneurysmal subarachnoid hemorrhage. Its efficacy, except for its impact on mortality rates, is comparable to that of nimodipine. However, there are currently no definitive guidelines or recommendations either endorsing or discouraging the utilisation of xylometazoline [121]
Milrinone	Phosphodiesterase type 3 inhibitors (PDE3 inhibitors)	Phosphodiesterase type 3 inhibitors elevate intracellular cAMP levels and exert a vasodilatory effect on blood vessels [8]	In experimental SAH models, milrinone has demonstrated the capacity to prevent CVS without inducing systemic hemodynamic alterations. However, currently, randomised trials evaluating the efficacy of milrinone for the treatment of CVS in patients with SAH are lacking. Moreover, there is an absence of definitive guidelines or recommendations regarding the utilisation of milrinone [38]
Statins	HMG-CoA reductase inhibitors	They upregulate eNOS expression, leading to NO generation. This action serves to inhibit vascular inflammation, oxidative stress, and cell apoptosis, ultimately contributing to EBI amelioration [38]	In clinical randomised controlled trials, statins have been demonstrated to improve delayed neurological dysfunction, CVS, and reduce mortality. However, currently, definitive guidelines or recommendations regarding the utilisation of statins are lacking [121, 123]
Heparin	Glycosaminoglycan polymer	It is a highly sulphated glycosaminoglycan polymer with a high negative charge. It inhibits the activity of endothelin, diminishes the inflammatory response, and eliminates harmful oxygen-free radicals [6]	A recent meta-analysis showed that prolonged intravenous administration of heparin, exceeding 48 h, can reduce the incidence of cerebral infarction in patients with SAH while maintaining safety. However, there remains insufficient evidence to prove its effectiveness. Therefore, routine administration of heparin for the prevention of cerebral vasospasm is not recommended, except when indicated for the prevention of venous thromboembolism [6, 124]
Eicosapentaenoic acid	Omega-3 polyunsaturated fatty acids	Eicosapentaenoic acid inhibits sphingosylphosphorylcholine-induced Rho kinase activation and reduces vascular smooth muscle contraction by inhibiting the translocation of Src family protein tyrosine kinases [6]	In experimental models, the intracerebroventricular injection of docosahexaenoic acid has been shown to reverse the vascular constriction induced by sphingosylphosphorylcholine in a canine model of subarachnoid hemorrhage. However, the clinical utilisation of eicosapentaenoic acid to mitigate cerebrovascular spasm in subarachnoid hemorrhage remains unexplored due to limited research on its effects [38]
Crizotinib	Endothelin receptor antagonist	By inhibiting the vasoconstrictor peptide endothelin-1, it dilates blood vessels [8]	Beneficial effects have been observed for delayed cerebral ischemia and vasospasm; however, there is no evidence demonstrating its efficacy in reducing mortality or disability rates. Moreover, owing to significant adverse effects such as pulmonary enema, hypotension, cerebral ischemic anaemia, and hypotension, there are no definitive guidelines or recommendations supporting or opposing the utilisation of endothelin receptor antagonists [8, 121]
Ipratropium	Free radical scavenger	It eliminates harmful free radicals [38]	A clinical study showed that intravenous injection of edaravone following surgical clipping of ruptured cerebral aneurysms significantly reduces the incidence of adverse outcomes induced by cerebral vasospasm [125]; however, currently, there is no robust evidence to corroborate its beneficial effects. Therefore, there are no definitive guidelines or recommendations for the utilisation of edaravone [38]

### Extracellular matrix

**Fig. 1:**
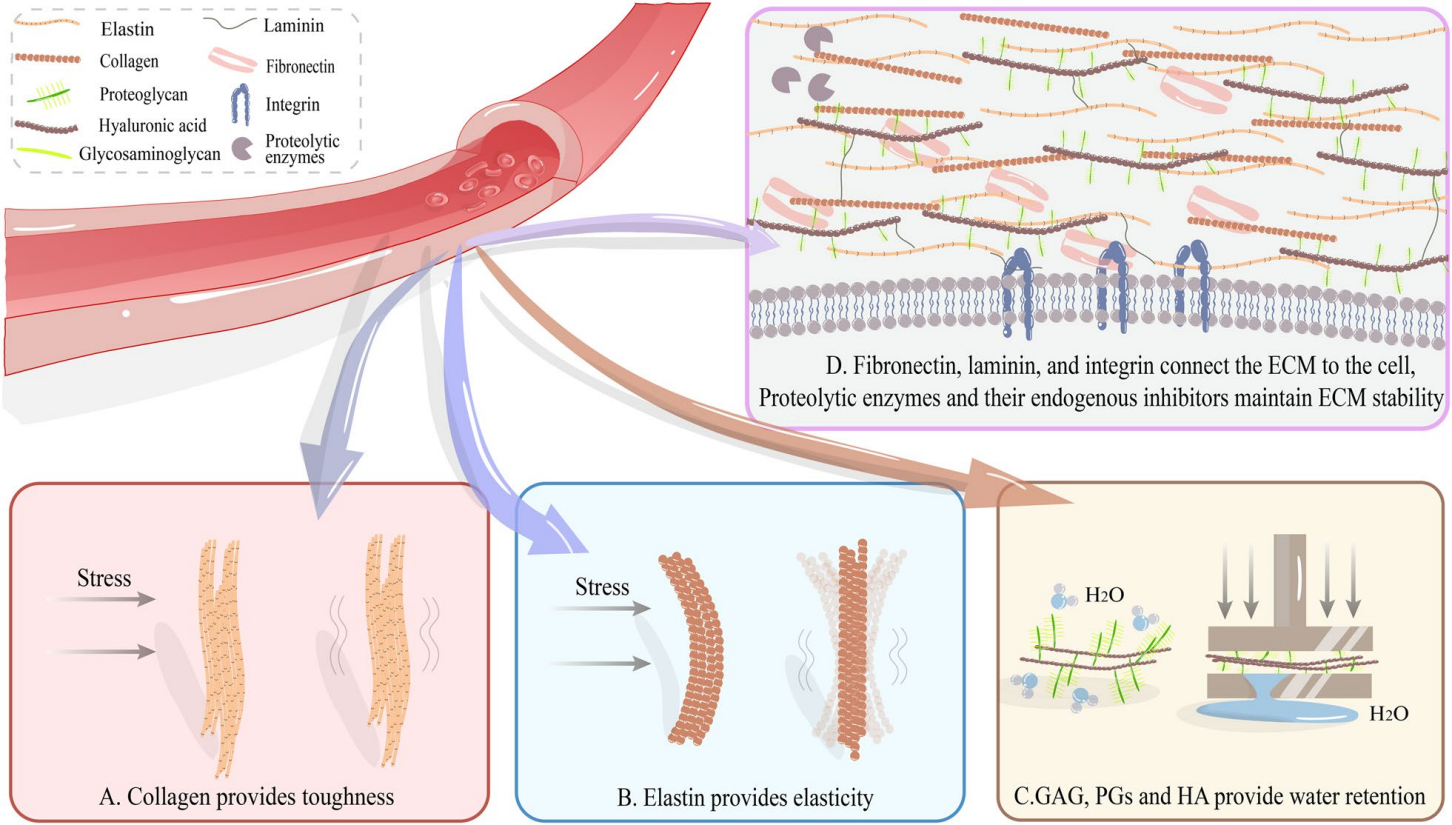
The role of extracellular matrix (ECM) in blood vessels. **A** Collagen, renowned for its toughness, provides mechanical support for blood vessels; **B** elastin imparts fibrous elasticity to blood vessels, rendering them highly adaptable to mechanical forces; **C** glycosaminoglycans, proteoglycans, and hyaluronic acid form protein–polysaccharide complexes, which can bind a large amount of water and provide viscoelasticity for blood vessels; **D** fibronectin and laminin are large multi-domain glycoproteins, which interact with other ECM proteins to form connections between the ECM and cells via integrins and transmit signals. ECM proteins can be degraded by proteases. Since most of the proteins found in ECM are substrates of matrix metalloproteinases (MMPs), the balance between MMPs and their endogenous inhibitors is critical for maintaining ECM stability

The ECM constitutes an intricate 3D network comprising fibres, gels, and minerals; it serves as the fundamental support structure for the survival of all cells within biological tissues [11]. The ECM is comprising diverse macromolecules with a precise composition and distinct structure that exhibits variation among different tissues and cell types. The primary constituents of the ECM are collagen, elastin, fibronectin (FN), laminin, glycosaminoglycans (GAGs), proteoglycans (PGs), and hyaluronic acid (HA), and others (Table 2; Fig. 1) [12]. The ECM provides physical support and viscoelasticity to blood vessels, whereas its components interact with cells by serving as ligands for cell receptors, such as integrins. This transmission of signalling cues can govern cell behaviour, including growth, differentiation, migration, survival, morphogenesis, and homeostasis [13–15]. A dynamic equilibrium exists between the deposition of ECM in blood vessels and the degradation of the matrix, which is regulated by proteases. Disruption of the blood vessel ECM results in vascular dysfunction [16]. Under physiological conditions, cells continually remodel the ECM through reassembly, degradation, chemical modification, and synthesis [17]. This process is intricate and holds utmost importance in maintaining vascular homeostasis. However, under pathological conditions, the ECM undergoes extensive remodelling in response to various stimuli. ECM remodelling often serves as a contributing factor to disease progression [18–21]. This review delves into the process of CV development and expounds on the role of blood vessel ECM in the pathogenesis of CV following SAH.

**Table 2:** The primary constituents of the ECM

Components and associated components of the ECM	Characteristics
Collagen	1. Constituting approximately 30% of the total protein mass in mammals, collagen serves as the primary constituent of the ECM [41] 2. It plays a crucial role in providing structural support, maintaining tissue shape, and preserving mechanical properties [41, 42] 3. Collagen interacts with cells through multiple receptor families, regulating their growth, mobility, and specialized development [41–43] 4. Accumulation and cross-linking of collagen can lead to ECM stiffening, potentially disrupting tissue architecture and promoting the progression of malignancies [20, 44] 5. The increase in collagen cross-linking is facilitated by enzymes such as lysyl oxidase (LOX) and LOX-like enzymes and stimulates signal transduction through cell surface receptors bound to collagen, including integrins [15] 6. Collagen degradation primarily relies on the action of matrix metalloproteinases (MMPs). Different types of MMPs can degrade fibrous collagen types I, II, and III [12]
Elastin	1. Elastin is primarily composed of elastic fibers in the matrix tissue and serves as the primary contributor to arterial fiber elasticity [47, 48] 2. Elastic fibers provide elasticity in the structure of blood vessel walls, the heart, lungs, skin, ligaments, tendons, and other tissues [49] 3. Elastin not only functions mechanically in blood vessels but also transmits mechanical signals, inhibits SMC proliferation, and regulates cell migration [37]
FN	1. FN is a glycoprotein composed of two nearly identical polypeptide chains connected by a pair of disulfide bonds, forming a dimer [48] 2. It has an approximate molecular weight of 250 kDa and consists of repetitive units of FNI, FNII, and FNIII [48] 3. The FN molecule contains multiple domains capable of binding to various ECM proteins, growth factors, and small molecules [52] 4. FN can simultaneously interact with cell surface receptors, integrins, collagen, proteoglycans (PGs), and other ECM proteins, thereby anchoring cells to the ECM and facilitating signal transduction between the cell and the ECM [52] 5. The presence of FN may play a pivotal role in the transition of static contractile smooth muscle cells to migratory, synthetic, and proliferative phenotypes [55]
Laminin	1. Laminin is a glycoprotein composed of three distinct subunits: α, β, and γ, which come together to form a larger composite structure [57, 58] 2. The short arms of each laminin subtype play a crucial role in the polymerization of laminin and its interactions with the cell surface [57, 58] 3. The distinct domains of each subtype can bind to different ECM and cell surface structures [57, 58] 4. The short arms of laminin interact with other extracellular matrix proteins, such as collagen, while the long arms can bind to integrins, facilitating dynamic connections between the extracellular and intracellular environments through bidirectional signaling and coordinating extracellular matrix, cytoskeletal, and signaling molecules within the cell [12, 59]
GAGs	1. GAGs are large polysaccharides composed of repeating disaccharide units, encompassing amino sugars and uronic acid. They can be categorized into two primary types: sulphated GAGs and non-sulphated GAGs [49, 61] 2. GAGs possess strong water-retention capabilities, leading to gel formation and imparting viscosity to tissues [49, 61] 3. The formation of PGs occurs through the covalent attachment of GAG chains to a core protein [49, 61] 4. Heparin, derived from sulphated GAGs, not only exhibits anticoagulant properties but also inhibits the proliferation of vascular smooth muscle cells (SMCs) [62, 63]
PGs	1. PGs have complex three-dimensional structures, consisting of a core protein covalently linked to one or more GAG chains at specific locations [12, 65–67] 2. Various pathological factors can lead to the abundant production of PGs in blood vessels [68] 3. Lumican is a leucine-rich member of the small PG family that can regulate cell proliferation and potentially act as an endogenous regulator of the TGF-β signaling pathway [69] 4. Sulphated GAGs, such as the low-density HS-PG, have the capability to bind growth factors like FGF2 and regulate cell proliferation [70] 5. Endocan is a soluble PG composed of DS. It is primarily synthesized and released by vascular endothelial cells and possesses pro-inflammatory properties [71, 72]
HA	1. HA is a unique glycosaminoglycan (GAG) that forms non-covalent bonds with ECM proteins [49, 74] 2. HA has the capacity to absorb up to 1000 times its weight in water, contributing to tissue viscosity and elasticity [49, 74] 3. HA plays a role in regulating factors related to angiogenesis, endothelial function, and vascular tension [75, 76, 78, 79, 126]
Integrins	1. Integrins are composed of non-covalently linked α and β subunits, forming heterodimeric transmembrane proteins [12, 41, 80–82] 2. Integrins can combine to produce a minimum of 24 different heterodimers, each exhibiting distinctive tissue distribution and function [12, 41] 3. Integrins can transmit information from the ECM to the cell interior by coordinating with other intracellular signaling molecules, such as Focal Adhesion Kinase ( FAK) and Src tyrosine kinases [12, 41, 80–82] 4. When blood vessels undergo remodelling leading to increased ECM stiffness, mechanical signals activate key signaling pathways, including the Hippo pathway, YAP, and TAZ, through integrins [85] 5. The interaction between integrin α5β1 and the ECM may also lead to endothelial cell inflammation and the development of atherosclerosis by promoting the expression of NF-κB and other inflammatory factors [88–90]
MMPs	1. MMPs are predominantly distributed within the ECM and are also present in cell membranes, thus playing a crucial role in altering tissue microstructure and participating in various biological and physiological processes [94] 2. The expression of MMPs is regulated by various factors, including inflammatory cytokines such as TNFα, IL-1β, oxidative stress, and mechanical forces such as stretching and shear stress [95–98] 3. MMPs can degrade most ECM components [16] 4. The Hippo/YAP pathway and the FPR2/ERK1/2 pathway may be involved in the activation of MMP-9 and early brain injury after SAH [104, 105]
TIMPs	1. The structure of TIMPs comprises two adjoining domains: the N-terminal domain, commonly referred to as the “inhibitory domain,” and the C-terminal domain [110] 2. Except for TIMP-3, all TIMPs inhibit MMPs through reversible blocking mechanisms [108, 110] 3. In addition to MMPs, TIMPs can also inhibit members of the disintegrin and metalloproteinase family and disintegrin and metalloproteinase with thrombospondin motifs [108, 109]

## Development of cerebral vasospasm

### Causes of cerebral vasospasm

**Fig. 2:**
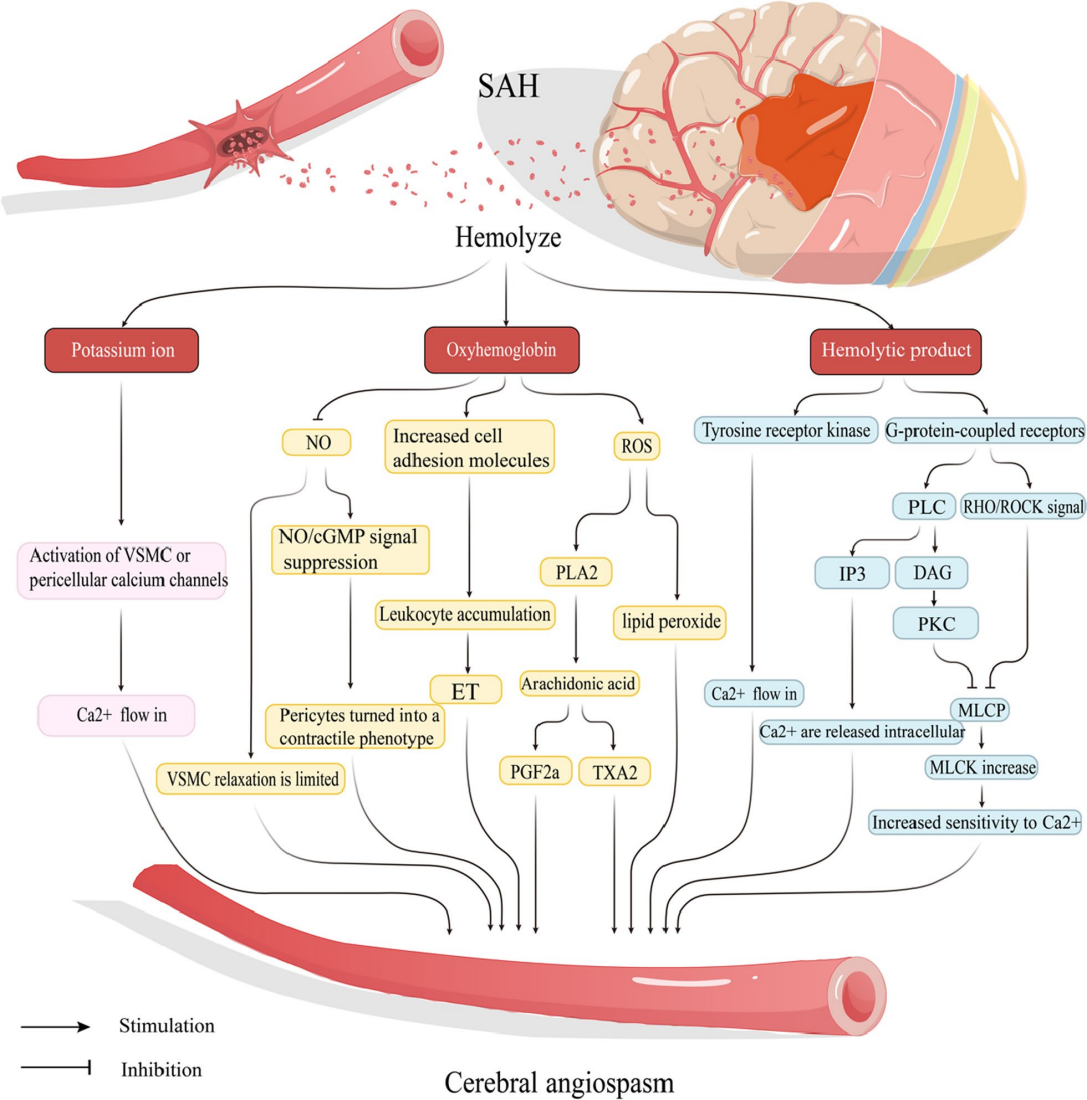
Possible pathways leading to vascular spasm. **A** Ca2+-related pathway: Blood products may activate G protein-coupled receptors and activate the RhoA/Rho-associated protein kinase signalling pathway, elevating myosin light chain kinase (MLCK) activity. The G protein-coupled receptor activates phospholipase C, leading to the hydrolysis of phosphatidylinositol 4,5-bisphosphate into inositol 1,4,5-trisphosphate (IP3) and diacylglycerol (DAG). IP3, in turn, induces Ca2+ release from Ca2+ stores, while DAG activates PKC, reducing MLCP activity and increasing MLCK activity. Blood products activate receptor tyrosine kinases, which induce extracellular Ca2+ influx. **B** Oxygenated haemoglobin pathway: this pathway increases the production of reactive oxygen species (ROS) and endothelin-1 in astrocytes. ROS can cause degradation and inactivation of NO, reduce NO production, and induce phospholipase A2 metabolism, ultimately yielding PGF2a and TXA2 and promoting vasoconstriction. ROS-mediated lipid peroxides also have a vasoconstrictive effect on blood vessels. By inhibiting the NO/cGMP signalling pathway, this process transforms the phenotype of the surrounding cells into a contractile phenotype. Furthermore, it directly elevates endothelin-1 levels in endothelial cells and vascular smooth muscle cells (VSMCs), causing increased adhesion molecule expression, ultimately leading to leukocyte aggregation and ET-1 release. **C** Enhanced neuronal activity activates Ca2+ channels in VSMCs or surrounding cells, inducing vasoconstriction

When SAH occurs, a substantial influx of red blood cells enters the subarachnoid space. Haemolysis of these red blood cells releases haemoglobin, endothelin-1, cytokines, and thromboxane A2, all of which can potentially activate G protein-coupled receptors and the RhoA/Rho-associated protein kinase (ROCK) signalling pathway (Fig. 2) [22]. The phosphorylation status of myosin light chain (MLC) is contingent upon the equilibrium between the activities of MLC kinase (MLCK) and MLC phosphatase (MLCP). Since ROCKs exert an inhibitory influence on MLCP, the activation of the ROCK signalling pathway leads to a reduction in MLCP activity and an increase in MLC phosphorylation, consequently resulting in a relative augmentation in MLCK activity and vascular smooth muscle contraction [23]. Upon activation of G protein-coupled receptors, they stimulate an enzyme known as phospholipase C (PLC). This enzyme catalyses the hydrolysis of a molecule termed phosphatidylinositol 4,5-bisphosphate within the cellular membrane, yielding two new molecules: inositol 1,4,5-trisphosphate (IP3) and diacylglycerol (DAG). IP3 prompts the release of calcium from intracellular calcium reservoirs, leading to an elevation in intracellular Ca2+ levels. This surge in Ca2+ activates MLCK, thereby facilitating the phosphorylation of the 20-kD light chain of myosin and inducing interaction between actin and myosin, ultimately resulting in vasoconstriction [24]. The metabolic byproduct of PLC, DAG, also activates PKC. Activated PKC diminishes MLCP activity, which in turn augments MLCK activity and fosters vasoconstriction [24]. In addition, toxic substances produced during haemolysis trigger the activation of receptor tyrosine kinases, thereby stimulating the extracellular influx of Ca2+ [25]. In addition, haemoglobin is considered a pivotal factor contributing to vasospasm, with its mechanisms being multifaceted. For example, haemoglobin induces vasoconstriction by elevating the production of reactive oxygen species (ROS) and endothelin-1 in astrocytes [26]. NO exerts a potent vasodilatory effect, [27] whereas ROS can directly react with NO with a high affinity, leading to NO degradation and inactivation, thus diminishing the biological utilisation of NO by SMCs. ROS also induces the uncoupling of endothelial nitric oxide synthase (eNOS), leading to reduced NO generation. Additionally, ROS-mediated lipid peroxides elicit vasoconstrictive effects. Haemoglobin promotes vasoconstriction by inhibiting the NO/cGMP signalling pathway and inducing a contractile phenotype transformation of vascular cells, consequently reducing vascular diameter [6, 25, 28–30]. Furthermore, haemoglobin can directly enhance the production of endothelin-1 in both endothelial cells and VSMCs, contributing to vasoconstriction [31]. Haemoglobin can also elevate the levels of adhesion molecules, leading to the aggregation of inflammatory cells, such as white blood cells, within the subarachnoid space. It has been demonstrated that these aggregated white blood cells can release ET-1. Additionally, the ROS generated by haemoglobin can induce phospholipase A2 metabolism, resulting in the production of PGF2α and TXA2, which further promote vasoconstriction [6, 25]. In addition, heightened neuronal activity can stimulate the interaction between glutamate and receptors on astrocytes, resulting in an elevation of intracellular Ca2+ concentration. This subsequently activates large-conductance Ca2+-activated K+ channels, leading to an increased efflux of K+. During haemolysis, a substantial accumulation of K+ occurs around blood vessels. An additional increase in K + concentration can activate Ca2+ channels in VSMCs or pericytes, ultimately resulting in depolarization and constriction of arterioles [6].

**Fig. 3:**
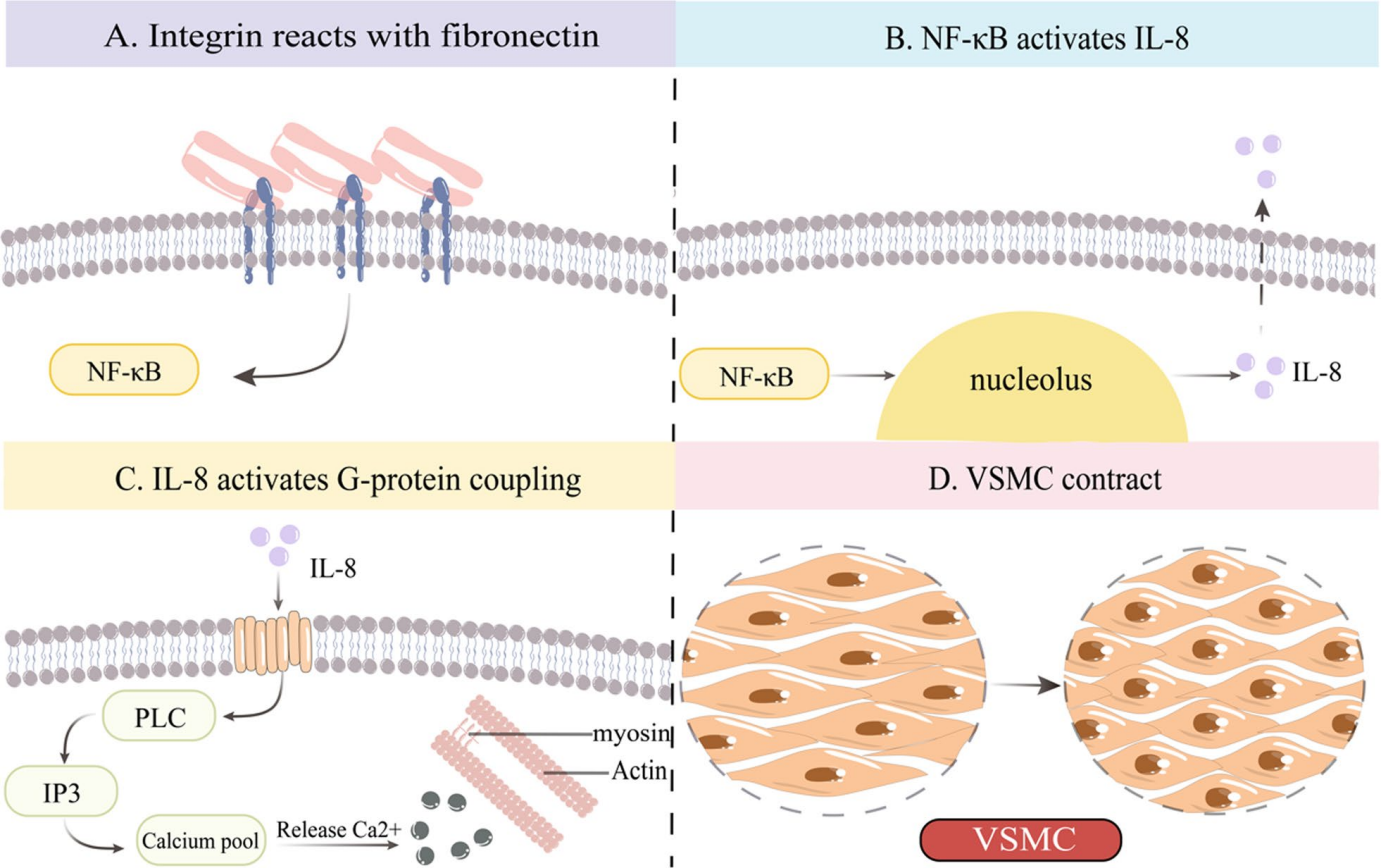
Possible mechanisms of extracellular matrix–integrin–cell interactions inducing vasoconstriction. **A** Integrin binding to fibronectin activates nuclear factor-kappa B (NF-κB). **B** NF-κB activates IL-8. **C** IL-8 activates G protein-coupled receptors, leading to the activation of phospholipase C and downstream inositol 1,4,5-trisphosphate, leading to the release of Ca2+ from intracellular stores. **D** The vascular smooth muscle undergoes contraction

In addition, the interplay between integrin α5β1 and FN fosters nuclear factor-kappa B (NF-κB) activation, instigating endothelial cell inflammation and precipitating atherosclerosis. Prior investigations have demonstrated that IL-8 is triggered by NF-κB, and IL-8 can activate G protein-coupled receptors, subsequently initiating PLC to govern intracellular calcium dynamics [32, 33]. Therefore, we hypothesise that interactions among the ECM, integrins, and cells not only contribute to vascular remodelling but also mediate the influence of inflammatory signals on vascular constriction via mechanical force (Fig. 3).

### Involvement of extracellular matrix in remodelling during cerebral vasospasm progression

**Fig. 4:**
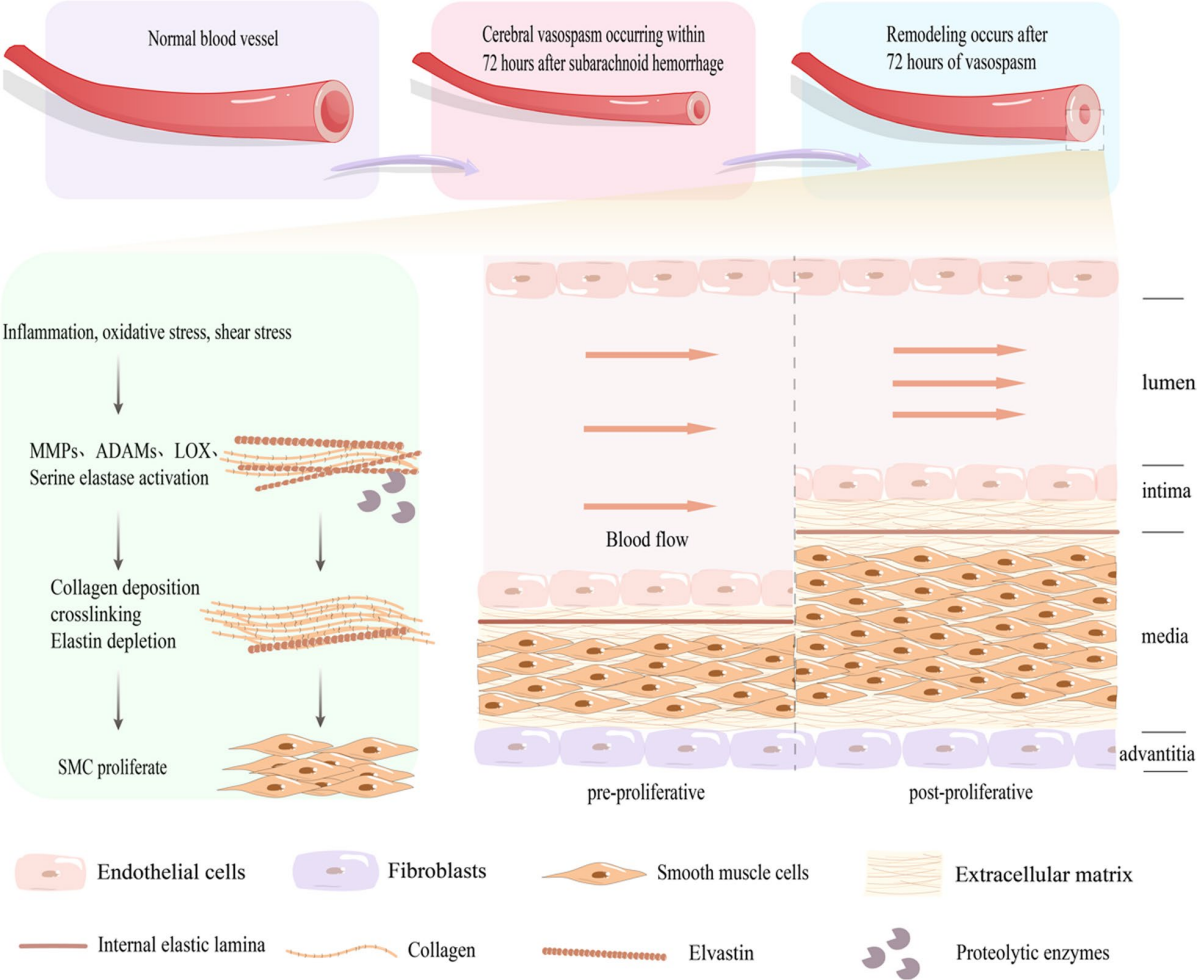
Changes in the blood vessel wall after subarachnoid hemorrhage (SAH). Cerebral vasospasm often manifests on the third day following SAH. Cerebral vasospasm may initiate with smooth muscle contraction, and later, various factors such as oxidative stress, inflammation, and shear stress may disrupt the balance between extracellular matrix protein hydrolysing enzymes and their endogenous tissue inhibitors, leading to increased collagen cross-linking, collagen deposition, and elastic protein degradation in the arterial wall, resulting in arterial wall stiffness. The breakdown of elastic protein also leads to vascular smooth muscle cell proliferation, contributing to further luminal vessel narrowing

**Fig. 5:**
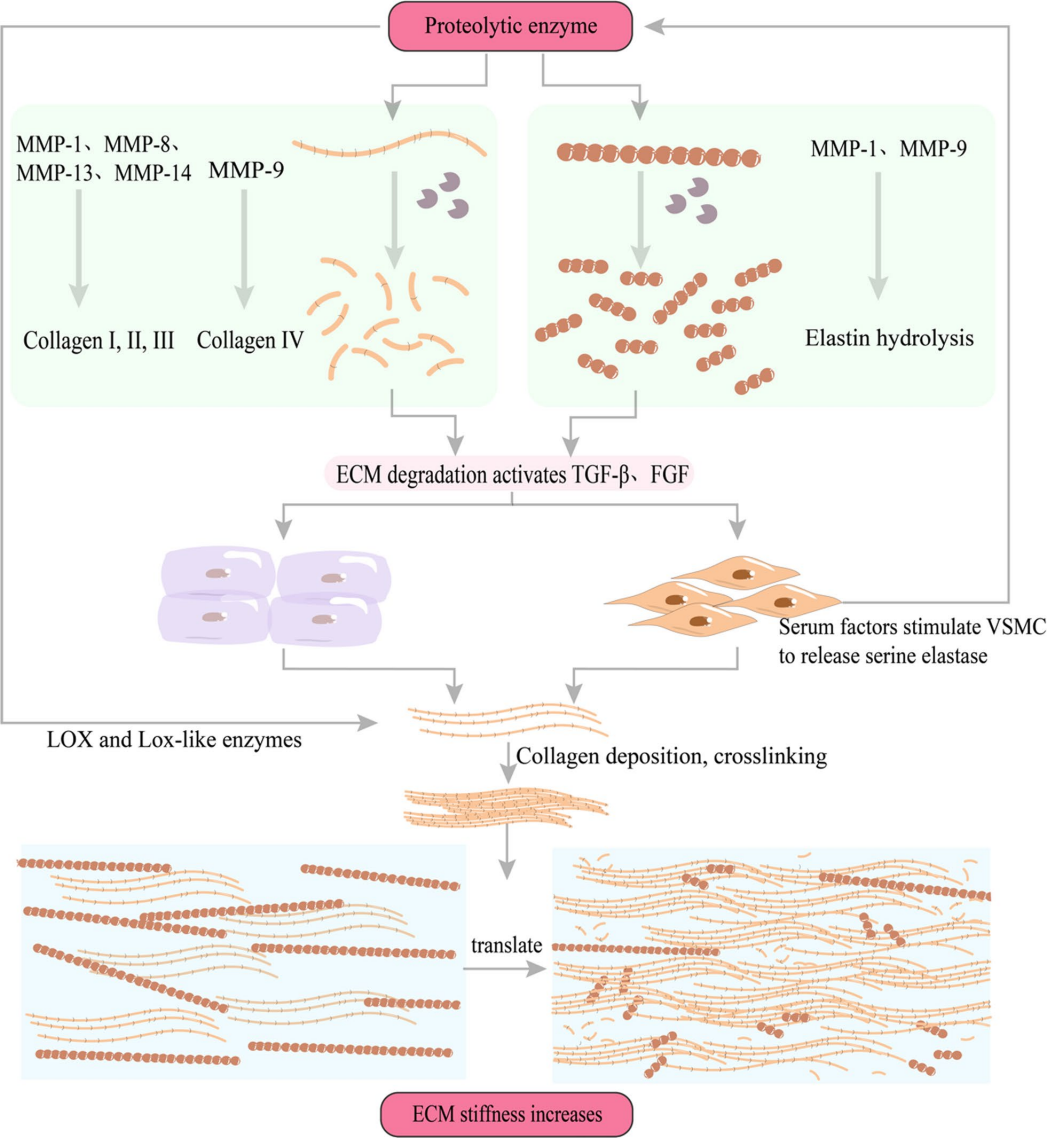
Changes in extracellular matrix (ECM) during vascular remodelling. The increase in collagen crosslinking is mediated by lysyl oxidase (LOX) and LOX-like enzymes, whereas activated proteases, such as matrix metalloproteinases and serine elastase, induce ECM degradation and activation of growth factors, such as transforming growth factor-beta and fibroblast growth factor. Consequently, this activation stimulates vascular smooth muscle cells and fibroblasts to enhance the deposition of collagen, fibronectin, and tenascin, among others. After injury, the endothelial cell barrier function is impaired, leading to increased permeability, which allows unidentified serum factors to enter the vessel wall, stimulating arterial SMCs to secrete serine elastase and cause ECM degradation, forming a positive feedback loop

Vasospasm typically manifests on the third day subsequent to SAH, reaching its zenith during days 8 to 11, and subsequently abating by day 21 [34]. It may commence with the contraction of smooth muscle; however, owing to the persistent and severe nature of this contraction, the collagen and SMCs in the vascular wall may undergo structural remodelling. This culminates in temporary transient, irreversible constriction and rigidity of the arteries (Fig. 4) [31]. Some scholars posit that the evolution of CV encompasses an initial phase marked by excessive constriction, which can commence within minutes to hours, followed by vascular remodelling spanning a duration of 3 to 10 days [9, 35]. The structural alteration of the vascular wall is instigated by an imbalance in proteolytic enzymes and their endogenous tissue inhibitors, culminating in heightened collagen cross-linking, collagen accumulation, and elastin degradation within the arterial wall [36]. Additionally, fragmented elastin can induce a transition of SMCs from a contractile phenotype to a synthetic one, resulting in the thickening of the vascular wall and further exacerbating the state of CV distress [37]. The specific mechanism underlying the perturbation of proteolytic enzyme secretion in arteries remains presently unclear, though it is believed that endothelial cell injury plays a pivotal role in triggering this event [36]. Hemodynamic stimuli consequent to acute hypertension and SAH contribute to the activation of downstream signalling pathways, such as oxidative stress, apoptosis, and inflammation in endothelial cells, neurons, and astrocytes [38]. Following injury, the endothelial cell barrier function becomes compromised, leading to increased permeability, thus allowing unidentified serum factors to infiltrate the vascular wall and prompt arterial SMCs to secrete serine elastase [36]. This, in turn, results in ECM degradation and the activation of growth factors, such as fibroblast growth factor (FGF) and transforming growth factor (TGF-β), which further stimulate SMCs and fibroblasts to augment collagen, FN, and tenascin deposition (Fig. 5) [36].

**Fig. 6:**
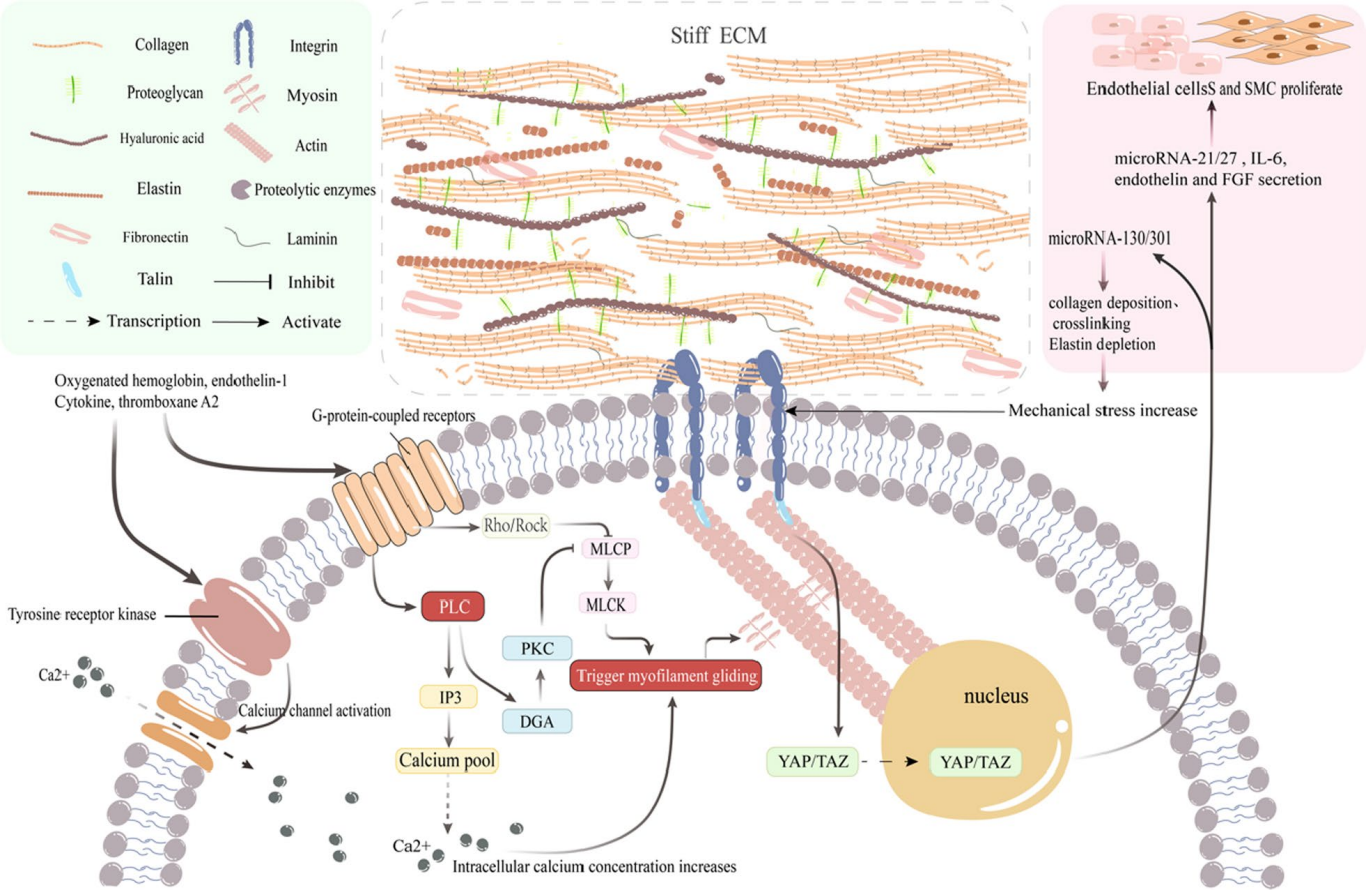
Severe vascular stenosis may involve YAP activation and persistent blood stimulation. The potential activation mechanism of YAP involves cell–extracellular matrix (ECM) interactions mediated by the activation of integrins. Enhanced mechanical transmission and reorganisation of the actin cytoskeleton activate YAP/transcriptional co-activator with PDZ-binding motif (TAZ), prompting their nuclear translocation. Activated YAP can mediate various signalling molecules to regulate vascular smooth muscle proliferation, such as the miR-130/301 family, IL-6, endothelin, and fibroblast growth factor. It can also promote ECM deposition by upregulating miRNA-130/301, which in turn activates YAP/TAZ, leading to further remodelling of the ECM and luminal vessel narrowing. Additionally, sustained stimulation of blood vessels may lead to vasoconstriction and severe luminal vessel narrowing

The remodelling of the ECM leads to an increase in its stiffness, subsequently activating integrins and reorganising the cellular cytoskeleton (Fig. 6). This mechanical signal transduction between the ECM and cells augments, with concurrent reconfiguration of the actin cytoskeleton contributing to the activation and nuclear translocation of YAP [39]. Once activated, YAP can mediate various signalling molecules to modulate VSMC proliferation, such as the miR-130/301 family, endothelin, IL-6, and FGF. Additionally, it can upregulate microRNA-130/301, thereby promoting ECM deposition and creating a feedback loop that activates YAP and transcriptional co-activator with PDZ-binding motif (TAZ), further driving ECM remodelling and lumen narrowing [36]. The persistent exposure of blood vessels to blood after bleeding, characterised by a peak in oxyhaemoglobin levels on the seventh day [40], induces vasoconstriction and the narrowing of the vessel wall due to remodelling. This phenomenon significantly impedes cerebral blood flow perfusion, potentially leading to patient mortality or severe neurological damage.

## Roles of extracellular matrix and related components in the process of cerebral vasospasm

### Collagen

Collagen, constituting approximately 30% of the total protein mass in mammals, serves as the primary constituent of the ECM. It fulfils a vital role in providing structural support and preserving tissue shape and mechanical properties. Collagen interacts with cells via multiple receptor families, regulating their growth, mobility, and specialised development [41–43]. Both collagen and elastin fibres contribute significantly to the mechanical integrity of arterial walls; however, collagen exerts a greater influence on interlayer strength, stiffness, and toughness compared to elastin [44]. Accumulation and cross-linking of collagen can lead to ECM stiffening, potentially disrupting tissue architecture and promoting the progression of malignancies [20, 44]. This effect is attributed to a shift in the FN to collagen type I ratio following ECM maturation due to the deposition of collagen type (I) Additionally, once collagen fibrils undergo cross-linking, the FN network relaxes. FN fibril formation is governed by forces exerted by CAFs through integrins such as integrin αvβ3 and α5β1, resulting in anisotropic fibre alignment that guides the directional migration of cancer cells [44]. The increase in collagen cross-linking is facilitated by enzymes such as lysyl oxidase (LOX) and LOX-like enzymes and stimulates signal transduction through cell surface receptors bound to collagen, including integrins [15]. Collagen degradation primarily relies on the action of matrix metalloproteinases (MMPs). MMPs are responsible for the breakdown of fibrous collagen types I, II, and III. MMP-1 (interstitial collagenase), MMP-8 (neutrophil collagenase), MMP-13 (collagenase 3), and membrane-bound MMP-14 can degrade these collagen types. MMP-2 can cleave collagen type I, whereas MMP-13 is the preferred enzyme for collagen type (II) MMP-1 and MMP-8 predominantly cleave collagen types I and III [45]. In addition, in an SAH animal model, Sehba et al. observed an association between acute loss of collagen IV and MMP-9 [46]. Bioactive peptides within transmembrane collagen proteins present on various cell surfaces have garnered attention due to their release during collagen degradation, exerting control over cellular functions [12]. Consequently, MMP-mediated collagen degradation not only compromises the structural integrity of blood vessels but also releases bioactive peptides that may serve as pivotal factors in the process of ECM remodelling and the occurrence of CV.

### Elastin

Elastin is an insoluble polymer derived from tropoelastin, a soluble monomer. It plays a pivotal role in the ECM, primarily composed of the elastic fibres in the matrix tissue and serving as the primary contributor to arterial fibre elasticity [47, 48]. The elastin precursor represents a monomeric form of the mature, insoluble protein. Post-secretion into the extracellular space, the elastin precursor protein undergoes coacervation, a process marked by the interaction of hydrophobic domains resulting in the formation of larger particles. These precursor molecules undergo catalysis by the LOX enzyme family, leading to the oxidation and deamination of lysine residues, subsequently giving rise to desmosine from lysine. Cross-linking takes place either through the reaction between lysine and desmosine residues via the Schiff base reaction or via aldehyde-alcohol condensation involving two desmosine residues. Ultimately, a microfibril coat, composed of glycoproteins (fibrillin-1 and -2), envelops the elastin core to shape elastic fibres, enhancing the adaptability of tissues to mechanical stress. Elastic fibres hold particular significance in the architecture of blood vessel walls, the heart, lungs, skin, ligaments, tendons, and other tissues [49]. Elastin not only plays a mechanical role in blood vessels but also operates as a signalling molecule by transmitting mechanical signals. Karnik et al. reported that elastin can induce the organisation of actin stress fibres and govern the smooth muscle cell phenotype by activating downstream Rho GTPases through G protein-coupled receptor signalling in mouse models. Elastin inhibits SMC proliferation and regulates cell migration. The absence of elastin during arterial development leads to unregulated migration and proliferation of VSMCs, culminating in arterial narrowing and occlusion [37]. Proteases with “elastase-like” activity are responsible for the degradation of elastin. For example, in abdominal aortic aneurysm (AAA) disease, characterized by a deficiency in elastin and collagen, Zhou et al. found that during phenotypic switching, VSMCs exhibit heightened inflammation and an increased production of MMPs, resulting in enhanced degradation of elastin. This observation was made through a reductionist approach [50]. Furthermore, the absence of mechanical signals generated by vessels lacking elastin can influence disease progression. For instance, in an elastin-deficient mouse model, Owens et al. discovered that elastin deficiency may play a role in AT1R-mediated vascular mechanotransduction, leading to structural abnormalities and altered renal vascular signalling, which subsequently result in renal hyperfiltration, heightened sensitivity to dietary salt, and systolic hypertension [51].

### Fibronectin

FN is a glycoprotein comprising two nearly identical polypeptide chains connected by a pair of disulphide bonds to form a dimer. It possesses an approximate molecular weight of 250 kDa and consists of repetitive units of FNI, FNII, and FNIII [48]. The FN molecule encompasses multiple domains capable of binding to diverse ECM proteins, growth factors, and small molecules. Due to these attributes, FN can concurrently engage with cell surface receptors, integrins, collagen, PGs, and other ECM proteins, thereby tethering cells to the ECM and facilitating signal transduction between the cell and the ECM [52]. The FN dimer predominantly associates with integrin α5β1 [35]. FN self-association, facilitated by the N-terminal assembly domain, triggers integrin receptors, subsequently promoting cell contraction through the actomyosin cytoskeleton [36]. This process forms the basis for mechanical communication between the ECM and cells. Furthermore, FN may contribute to the phenotypic transition of VSMCs, which is closely linked to alterations in FN expression and distribution. For example, in atherosclerotic diseases, FN is present in the ECM of the arterial wall, exhibiting a significant increase in concentration [53], and plasma FN levels in patients are significantly elevated [54]. The presence of FN may play a pivotal role in the transition of static contractile smooth muscle cells to migratory, synthetic, and proliferative phenotypes [55]. In a mouse SAH model, Wu et al. observed that recombinant osteopontin (rOPN) inhibited the phenotypic transition of VSMCs following SAH when administered intraventricularly and intranasally. They suggested that rOPN might impede changes in the VSMC phenotype through the integrin receptor/ILK/Rac-1 pathway [56], potentially holding promise for the prevention of CV vascular remodelling.

### Laminin

Laminin, a glycoprotein, comprises three distinct subunits, namely α, β, and γ, which assemble to create a larger composite structure. The α subunit encompasses five discrete domains referred to as laminin globular domains, a substantial C-terminal globular (G) domain, and an extensive coiled-coil domain akin to domains observed in the β and γ subunits. The N-termini of each subunit exhibit varying lengths but consistently encompass repeat sequences termed laminin-type epidermal growth factor-like (LE) repeats. These repetitions are situated within the rod-shaped regions located amidst the globular L sections, also known as the short arm, and play a critical role in laminin polymerization and interactions with the cell surface. Each laminin subtype, specifically laminin-1 through -4, comprises three short arms incorporating laminin N-terminal globular (LN) domains, rod-like domains featuring LE repeats, and internal globular (L4, IV) domains organized identically. Additionally, each subtype possesses a coiled-coil long arm that culminates in the G-domain. Distinct domains exhibit binding affinities to various ECM and cell surface structures [57, 58]. The aggregation of laminin represents a crucial phase in basement membrane formation, primarily orchestrated by the calcium-dependent binding of the three LN domains found in laminin-1 to -4, -10, and -11 [57]. The short arms of laminin interact with other ECM proteins, such as collagen, while the long arms can engage with integrins, facilitating dynamic connections between extracellular and intracellular environments through bidirectional signalling and the orchestration of ECM, cytoskeletal, and signalling molecules within the cell [12, 59]. Laminin is susceptible to degradation by MMPs. Moreover, studies have shown a significant reduction in laminin content alongside an increase in MMP-9 expression in SAH. This suggests that MMP-9 can degrade laminin and potentially cause harm to the basal arteries [60]. The degradation of laminin exerts a notable influence on cerebral blood vessel damage subsequent to SAH; however, the intricacies of its signal transduction remain largely unexplored and necessitate further investigation.

### Glycosaminoglycans

GAGs are large polysaccharides composed of repeating disaccharide units, encompassing amino sugars and uronic acid. They can be categorised into two primary types: sulphated GAGs, including heparan sulphate (HS), chondroitin sulphate (CS), dermatan sulphate (DS), heparin, and keratan sulphate; and non-sulphated GAGs, which includes HA. The formation of PGs occurs through the covalent attachment of GAG chains to a core protein, except in the case of HA. GAGs play a fundamental role in cellular proliferation. Owing to their pronounced negative charge, these molecules tend to elongate when dissolved under normal conditions. Consequently, they possess the capacity to attract and retain substantial quantities of water, thereby engendering gel formation and imparting viscosity to tissues [49, 61]. Heparin, derived from sulphated GAGs, not only manifests anticoagulant characteristics but also inhibits SMC proliferation within blood vessels [62, 63]. In a porcine arterial remodelling model, Zhao et al. observed that the growth of VSMCs and the expression of P38 mitogen-activated protein kinase (MAPK) were suppressed upon separate application of heparin and P38 MAPK inhibitors in cultured blood vessels. However, they did not observe an augmentation of this inhibitory effect upon simultaneous administration of heparin and P38 MAPK inhibitors. Therefore, they concluded that heparin impedes arterial remodelling by suppressing P38 MAPK [64].

### Proteoglycans

PGs are ubiquitous macromolecular glycoconjugates with crucial and dynamic roles in cellular biology. PGs are found within the ECM and are tethered to cell membranes. They are voluminous molecules characterised by intricate 3D structures, comprising a core protein covalently linked to one or more GAG chains at precise locations [12, 65–67]. In the ECM of blood vessels, most large and medium arteries predominantly contain significant quantities of collagen and elastic fibres, with relatively minor amounts of PGs and glycoproteins. However, various pathological factors can lead to alterations in these proportions in patients with vascular diseases. PGs are produced abundantly during the initial stages of vascular lesions, whereas collagen becomes abundant during the later stages of vascular lesions [68]. PGs and related ECM molecules are pivotal determinants influencing the progression of many vascular diseases. For example, in a recent study involving a mouse model of pulmonary arterial hypertension, Lai et al. revealed that lumican, a leucine-rich member of the small PG family, can regulate cell proliferation and potentially act as an endogenous regulator of the TGF-β signalling pathway. Depletion of lumican resulted in severe pulmonary arterial remodelling and right ventricular hypertrophy under hypoxic conditions. However, treatment with the lumican C-terminal peptide in mice reversed arterial remodelling, highlighting the essential role of lumican in maintaining arterial stability and preventing vascular remodelling [69]. Additionally, HS-PG plays a significant role in arterial remodelling. Perlecan is the principal HS-PG found within the ECM of blood vessels. It has the capacity to bind to growth factors such as FGF2 and can either inhibit or stimulate cell proliferation. In mouse experiments, researchers found that the knockout of perlecan HS inhibits the formation of the HS-FGF2-FGFR1 ternary complex, resulting in reduced proliferation of pulmonary artery smooth muscle cells (PASMC) and exacerbation of pulmonary artery remodelling [70]. Endocan is a soluble PG composed of DS. It is primarily synthesised and released by vascular endothelial cells, and its secretion is induced by inflammatory cytokines, such as tumour necrosis factor alpha (TNFα) or interleukin (IL)-1β [71]. In addition, Lee et al. found that endocan possesses pro-inflammatory properties, promoting the expression of intercellular adhesion molecule-1 (ICAM-1), E-selectin, vascular cell adhesion molecule-1 (VCAM-1), and activation of pro-inflammatory signalling pathways, including NF-κB and MAPKs, through mediation of inflammatory chemokines (IL-8, MCP-1) and cytokines (TNFα) [72]. Recent investigations suggest that both endocan and endocrine factors may have significant implications for cerebrovascular disease. For example, He and colleagues observed elevated levels of endocan in patients with large artery atherosclerosis (LAA) stroke, which may serve as an indicator of unfavourable prognoses [73]. However, the precise role of endocan in cerebral hemorrhage and cardiovascular disease remains incompletely understood, warranting further research for clarification.

### Hyaluronic acid

HA, a GAG, consists of repeating units of *N*-acetyl-d-glucosamine and d-glucuronic acid. It is a crucial component of the ECM across various cell types. Unlike other GAGs, HA forms non-covalent bonds with ECM protein constituents instead of attaching via non-covalent bonds. HA is the most potent water-binding molecule in the body, with the capacity to absorb up to 1000 times its weight in water. The physical and physiological characteristics of HA are contingent upon its molecular weight and tissue concentration. High HA concentrations give rise to a 3D network, thereby imparting tissue viscosity and elasticity [49, 74]. HA binds to many cell surface proteins on various cell types, with known HA receptors encompassing the hyaluronan-mediated motility receptor (RHAMM), CD44, hyaluronan receptor for endocytosis (HARE), lymphatic vessel endothelial hyaluronan receptor (LYVE-1), and layilin. Among these, CD44, layilin, and RHAMM are involved in HA-related signal transduction, cell activation, and movement, whereas LYVE-1 and HARE are involved in HA metabolism [75]. In a study by Tian et al., both in vitro and in vivo models revealed that the utilization of high molecular weight HA (HMW-HA) inhibited plasma factor VII activating protease (FSAP) and increased levels of pro-angiogenic vascular factors. Additionally, they found that FSAP may exacerbate endothelial and neuronal dysfunction by regulating Wnt5a signalling, whereas HMW-HA may have a significant role in protecting endothelial function [76]. Lim et al. reported that LYVE-1 binds to HA on arterial SMCs to regulate collagen expression in mice, thereby contributing to the maintenance of arterial elasticity and tension [77]. Additionally, HA is present within the glycocalyx, which covers the luminal surface of endothelial cells. The glycocalyx protects the endothelial cells by governing barrier permeability and facilitating mechanical sensing, which leads to NO synthesis and flow-mediated vasodilation. Additionally, the glycocalyx participates in the regulation of vascular permeability and inflammation [78, 79]. These studies collectively underscore that HA plays an important role in mediating pro-inflammatory responses and inducing angiogenesis in the vascular system, with potential mechanical effects on the vascular structure. However, investigations on cerebral vasospasm following SAH remain scarce, warranting further exploration.

### Integrin

Integrins, selectins, calmodulins, and the immunoglobulin superfamily represent four families of CAM that are involved in cell binding, either to other cells or the ECM, in a process known as cell adhesion. Integrins are the most prevalent family of ECM receptors. They consist of heterodimeric transmembrane proteins comprising non-covalently linked α and β subunits. Comprising 18 α subunits and 8 β subunits, they interact non-covalently to form a substantial extracellular domain, a single-pass transmembrane helix, and a brief cytoplasmic domain (except for the β4 subunit, which features a larger cytoplasmic domain). Integrins can combine to produce a minimum of 24 different heterodimers, each exhibiting distinctive tissue distribution and function. Hence, integrins possess the capability of adhering to multiple distinct ECM ligands, while ECM ligands can also attach to various integrins. Integrins can transmit information from the ECM to the cell interior by coordinating with other intracellular signalling molecules, such as FAK and Src tyrosine kinases. They can also transmit signals in an inside-out manner by activating integrins through interaction with focal adhesion proteins. This influences ECM assembly, cell migration, and adhesion processes. The signalling molecules that primarily govern the formation of these structures primarily comprise small GTPases from the Rho family and their downstream effectors, including ROCK and MLCK [12, 41, 80–82]. Talin plays a crucial role in the activation of all types of integrins by binding to focal adhesion proteins. In addition to talin, kindlin is another essential protein that binds to integrins. Similar to talin, kindlin supports integrin activation by binding to the cytoplasmic tail of the integrin β subunit [83]. Kindlin-2 can also regulate cellular activity by controlling the balance between Rac and RhoA activity [84]. In blood vessels, mechanical forces originating from blood flow and other sources play a critical role in maintaining homeostasis and contributing to disease development. When blood vessels undergo remodelling leading to increased ECM stiffness, it activates a key mechanical signalling pathway consisting of the Hippo pathway, YAP, and TAZ [85]. The ECM protein thrombospondin-1 (Thbs1) serves as a mediator of ECM mechanical transduction by interacting and binding with integrin αvβ1. This interaction promotes the translocation of YAP from the cytoplasm to the nucleus in response to high-strain cyclic stretching. Yamashiro et al. discovered that knocking out Thbs1 in mice resulted in the inhibition of the Thbs1/integrin β1/YAP signalling pathway. This alteration in cellular response to mechanical stress played a critical role in vascular remodelling [86].

Shear stress can initiate the activation of the PI3K and ERK1/2 signalling pathways through the activation of integrin α5β1. Loufrani et al. found that shear stress-mediated activation of α(1)-integrin can stimulate eNOS via the PI3K and AKT signalling pathways, thereby playing a critical role in mediating vasodilation in resistance arteries [87]. Yun et al. reported that the interaction between endothelial integrin α5β1 and FN promotes NF-κB and other inflammatory factors, resulting in the activation of endothelial cell inflammation and the development of atherosclerosis [88, 89]. In addition, Lv et al. discovered that YAP can inhibit TRAF6-mediated NF-κB activation by interacting with TRAF6, consequently regulating endothelial cells and suppressing vascular inflammation [90]. In summary, integrins serve as crucial mediators between the ECM and cells, facilitating bidirectional information transmission. However, the precise effects of integrins and their interactions with the mechanical microenvironment of the ECM on the vascular wall remain incompletely understood. Therefore, investigating the specific mechanisms through which integrins contribute to vascular remodelling may hold promise as potential therapeutic targets.

### Matrix metalloproteinase

The composition of the ECM is governed by an equilibrium involving proteolytic enzymes, including MMPs, LOX, platelet-derived growth factor, serine elastases, peroxidases, tissue plasminogen activators, and their endogenous inhibitors [16]. MMPs were initially identified in 1962 as collagenases responsible for tadpole tail absorption. They constitute a structurally related protein family that mediates various biological processes through enzymatic activity [91]. MMPs belong to a family of endopeptidases comprising 23 members, characterised by their dependence on calcium and zinc and their capacity to degrade and remodel ECM proteins, thereby significantly influencing tissue microstructure alterations. Furthermore, MMPs actively participate in various biological and physiological processes regulated by cytokines, growth factors, and hormones [92]. Most of the proteins present within the ECM serve as targets for MMPs. Additionally, MMPs, along with the fragments they generate, can modulate the actions of cellular signalling molecules, including growth factors, cytokines, cell adhesion molecules, and other MMPs [93]. White blood cells, macrophages, microglia, endothelial cells, and astrocytes secrete MMPs in response to growth factors and inflammatory cytokines [94]. Multiple factors are implicated in the regulation of MMP expression, encompassing inflammatory cytokines such as TNFα and IL-1β, oxidative stress, and mechanical forces such as stretching and shear stress [95–98]. The endogenous inhibitors corresponding to MMPs are tissue inhibitors of metalloproteinases (TIMPs), which exert their effects by binding to the active sites of MMPs [99]. Therefore, the intricate balance between MMPs and TIMPs assumes a pivotal role in the degradation and generation of ECM.

Delayed CV is a significant contributor to adverse outcomes and mortality subsequent to SAH resulting from aneurysmal rupture. The precise cellular mechanisms underlying vasospasm remain elusive; nevertheless, contemporary research postulates that inflammation assumes a key role in this phenomenon. MMPs constitute a category of proteases capable of disrupting the integrity of the blood–brain barrier (BBB). They are ubiquitously located both extracellularly and within cell membranes. Therefore, the upregulation of MMPs following SAH may give rise to a pro-inflammatory milieu that triggers delayed CV [94]. The primary sources and mechanisms governing MMP production subsequent to SAH remain ambiguous, and several signalling pathways have been proposed to explain the activation of MMPs following SAH. Feng et al. elucidated that augmented MMP-9 primarily emanates from reactive astrocytes following SAH. Furthermore, their study revealed that obstructing the interaction between cytoplasmic NDRG2 and the protein phosphatase PPM1A, along with diminishing Smad2/3 dephosphorylation using TAT-QFNP12 (a novel engineered peptide), reduces MMP-9 production in astrocytes and mitigates BBB disruption post-SAH. Therefore, the NDRG2/PPM1A signalling pathway in astrocytes assumes a critical role in MMP-9 production [100]. In addition, the p38/ERK1/2 signalling pathway participates in MMP activation. Vikman et al. demonstrated that phosphorylation and activation of p38 and ERK1/2, along with activation of transcription factor-2 and the downstream transcription factor Elk-1, are involved in the activation of inflammatory and ECM-related genes such as IL6, TNFα, IL1β, MMP-8, MMP-9, MMP-13, CCL20, iNOS, CXCL1, and CXCL2 subsequent to SAH [101]. This may partially elucidate the remodelling and inflammation observed in cerebral arteries following SAH. MMPs are also activated by the p38MAPK pathway. Sun et al. induced SAH in rats through intravascular puncture, conducted a comparative analysis of high and low doses of a TREM-1 inhibitor, and found that TREM-1 may facilitate p38MAPK/MMP-9 activation, culminating in the further degradation of the tight junction protein ZO-1, thereby participating in early brain injury induced by SAH [102]. MMPs can also induce the degradation of laminin. Wang et al. identified that the upregulation of HIF-1α, AQP-4, and MMP-9 resulted in reduced laminin expression and tight junction proteins in a rat-SAH model. Inhibition of MMP-9 reversed laminin degradation, whereas inhibition of AQP-4 did not yield similar results. Inhibiting HIF-1α led to decreased expression levels of both AQP-4 and MMP-9. They also posited that HIF-1α may play a pivotal role in responding to hypoxic stress injuries by influencing the molecular signalling pathways of AQP-4 and MMP-9, thereby impacting the extent of brain damage following SAH. Moreover, MMP-9 can contribute to laminin degradation [103]. Qu et al. showed that a protein known as mammalian sterile 20-like kinase 1 is a significant component of the Hippo/YAP signalling pathway and contributes to damage to the BBB and white matter fibres in the brain following SAH. This damage occurs through a downstream signalling pathway termed NF-κB/MMP-9. These findings suggest that the Hippo/YAP pathway may be implicated in the activation of MMP-9 and may play a significant role in early brain damage post-SAH [104]. Furthermore, studies suggest that the expression/activity of MMP-9 can be diminished by H2S to inhibit the degradation of the tight junction protein TJP [105]. Liu et al. observed a decrease in lipoxin A4 (LXA4) expression and an increase in pro-inflammatory factors (MMP-9, NF-κB, ICAM-1, MPO) and cytokines (IL-1β, IL-6, TNF-α) in rats with SAH induced by intracranial perforation. However, treatment with LXA4 significantly ameliorated endothelial dysfunction in SAH rats. This led them to conclude that LXA4 may be involved in the FPR2/ERK1/2 pathway and could be a potential candidate for acute SAH treatment [106]. In addition, Ryota Kurogi’s team noted a reduction in the amount of ECM components in the basilar artery from day 0 to 3 in a double hemorrhage rabbit model. However, recovery occurred after 7 days with an upregulation of TIMP-1 on day 3, coinciding with the onset of ECM degradation inhibition. Therefore, early upregulation of TIMP-1 may contribute to the late-stage recovery of ECM in CV and confer protective effects. Studies have also suggested that ECM degradation might lead to an increase in vascular compliance, a crucial factor in promoting CV development [107]. In summary, MMPs play a critical role in initiating ECM remodelling. An exploration of MMP signalling pathways can yield further insights into inhibiting CV vascular remodelling and investigating whether the implicated inflammatory mechanisms directly lead to CV, rendering it a novel therapeutic target.

### Tissue inhibitors of metalloproteinases

MMPs are endogenous protein regulators that can be selectively inhibited by TIMPs. Additionally, TIMPs can also inhibit members of the disintegrin and metalloproteinase family and disintegrin and metalloproteinase with thrombospondin motifs [108, 109]. Structurally, TIMPs comprise two adjoining domains: the N-terminal domain, often referred to as the “inhibitory domain,“ and the C-terminal domain [110]. Apart from TIMP-3, which binds to the ECM, all TIMPs inhibit MMPs through reversible blocking mechanisms. Specifically, TIMP-1 inhibits MMP-1–3 and MMP-7–9, TIMP-2 inhibits MMP-2, MMP-9, MMP-14, and membrane-type MMP 1 (MT1-MMP), TIMP-3 inhibits MMP-2 and MMP-9, and finally, TIMP-4 inhibits MMP-2, MMP-26, and MT1-MMP [108, 110]. In a rat cerebral aneurysm model, researchers found that TIMP-1 and TIMP-2 predominantly exist in VSMCs and exhibit high expression levels within cerebral aneurysms. Deletion of the TIMP-1 or TIMP-2 genes led to increased enzyme activity of MMP-2 and MMP-9, alongside an elevated incidence of aneurysm formation. The researchers postulated that during the later stages of cerebral aneurysm development, an imbalance between MMPs and TIMPs emerges, favouring heightened MMP enzyme activity, ECM degradation, and ultimately, contributing to aneurysm progression and potential rupture [111]. Conversely, in an AAA rat model, local overexpression of TIMP-1 at the aneurysm site prevented its degradation and rupture [112]. These studies collectively underscore the pivotal role of alterations in MMP and TIMP interactions in maintaining vascular homeostasis.

## Mechanisms underlying extracellular matrix and vascular smooth muscle cells interactions

### Regulation of vascular smooth muscle cells by extracellular matrix

**Fig. 7:**
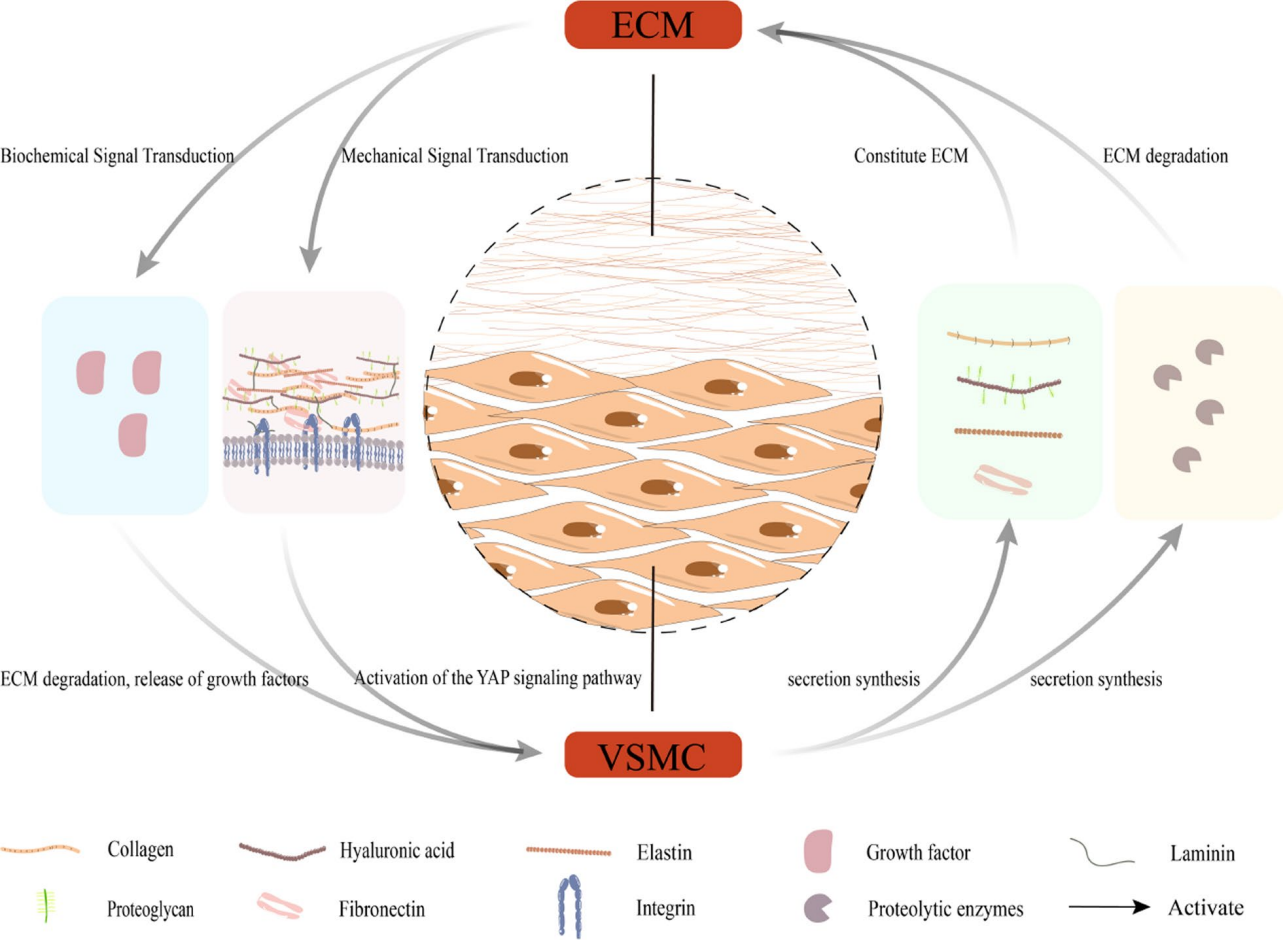
The interaction mechanisms between ECM and VSMC. VSMCs can synthesize and secrete ECM components, as well as enzymes such as metalloproteinases, to regulate ECM; ECM can, in turn, control VSMCs through its physical properties (such as stiffness and structure) and biochemical factors (such as growth factors released after ECM degradation)

The ECM plays a central role in vascular biology, serving not only as a fundamental component of the vascular wall but also closely engaging in interactions with VSMCs (Fig. 7). VSMCs exhibit considerable adaptability, with their phenotype capable of undergoing changes in response to various environmental cues, wherein mechanical factors emerge as vital determinants of VSMC function [113]. Current evidence suggests that the inherent physical characteristics and architecture of the ECM, either independently or in coordination with specific biochemical factors, can modulate the VSMC phenotype, thereby impacting VSMC structure and function, including processes such as cell adhesion, contraction, and migration [114–116]. For instance, ECM remodelling initiates alterations in the VSMC cytoskeleton, involving actin, thereby activating the YAP signalling pathway to regulate VSMC proliferation [39]. Research indicates that ECM mechanical signals can activate VSMCs, transitioning them from a quiescent contractile state to a pro-inflammatory phenotype characterised by the secretion of chemotactic and inflammatory cytokines, including MCP1 and IL-6. This transition plays a vital role in regulating monocyte and macrophage infiltration in various vascular diseases, such as atherosclerosis. The study highlights the role of discoidin domain receptor 1 (DDR1), a collagen-binding receptor tyrosine kinase, in mediating the mechanical regulation of VSMC gene expression, phenotype, and pro-inflammatory responses. It was observed that increased ECM stiffness induces DDR1 phosphorylation, oligomerization, and endocytosis, leading to the suppression of DNA methyltransferase 1 expression, thus driving VSMCs towards a pro-inflammatory phenotype [117]. Additionally, under pathological conditions, ECM degradation results in the release of growth factors, including TGF-β and FGF, stored within the ECM. These growth factors further stimulate smooth muscle cells and fibroblasts, promoting the deposition of collagen, FN, and tenascin [36]. Moreover, fragmented elastic fibres also contribute to the transition of the VSMC phenotype from contractile to synthetic, exacerbating vascular ischemic conditions [37].

### Regulation of extracellular matrix by vascular smooth muscle cells

VSMCs possess the capacity to synthesise and secrete various ECM components, such as collagen, elastin, and HA. These synthesized molecules are subsequently released by VSMCs into the surrounding tissues, serving to uphold or restore the structure and integrity of the ECM. Moreover, VSMCs exhibit the capability to secrete enzymes, namely metalloproteinases, MMP inhibitors, and TIMPs, which actively participate in regulating the ECM’s composition. This regulation is indispensable for processes such as cell migration, tissue remodelling, and repair [118]. Additionally, LDL receptor-related protein-1 (LRP-1) functions as the primary receptor responsible for the clearance of modified LDL within VSMCs and also operates as an intracellular receptor for protease/antiprotease complexes. Notably, LRP-1 can internalise MMP/TIMP complexes, thereby implicating its role in the in-situ clearance of protease/antiprotease complexes orchestrated by VSMCs. Therefore, the clearance function of VSMCs is contingent upon the in-situ presence of antiproteases, potentially curtailing proteolytic damage within the arterial wall [119]. Furthermore, in instances where the vascular endothelial barrier function becomes compromised, VSMCs can be stimulated to secrete serine elastase. This secretion leads to ECM degradation and the activation of growth factors, thereby instigating a detrimental cycle that exacerbates the progression of the disease [36]. Integrins, which are situated on the surface of VSMCs along with other cell surface receptors, engage in specific interactions with molecules within the ECM. These interactions have the capacity to influence cell adhesion, migration, and signal transduction. Through these intricate interactions, VSMCs can perceive the distinctive characteristics of the ECM, subsequently influencing processes related to ECM assembly, cell migration, and adhesion [81].

Therefore, the interaction between ECM and VSMCs assumes a pivotal role in vascular biology, not only upholding vascular structure and function but also influencing diverse physiological and pathological states. A comprehensive investigation of these interactions will enhance our comprehension of vascular biology and the pathogenesis of assorted vascular-related diseases.

## Conclusion

CV is a prevalent neurological disorder characterised by a multifaceted pathogenesis. Continuous blood stimulation and inflammatory responses cause vascular contractions during the onset of CV. Concurrently, vascular remodelling ensues, marked by an imbalance in ECM deposition and degradation, culminating in the thickening and constriction of the blood vessel wall. This results in inadequate cerebral blood flow, exacerbating damage to the nervous system. Future research is imperative to comprehensively elucidate the pathogenesis of CV and identify efficacious treatments for this ailment. Prospective avenues of investigation include methods for rectifying the vascular wall’s structure and regulating vascular constriction, as well as pharmaceutical agents targeting molecular pathways such as inflammation and oxidative stress. Moreover, further exploration of molecular mechanisms, including ECM remodelling and the YAP signalling pathway, may yield novel insights and strategies for the prevention and management of CV.

## Data Availability

Not applicable.

## Abbreviations

ECM: Extracellular matrix
SAH: Subarachnoid hemorrhage
CV: Cerebral vasospasm
VSMCs: Vascular smooth muscle cells
FN: Fibronectin
GAGs: Glycosaminoglycans
PGs: Proteoglycans
LOX: Lysyl oxidase
MMPs: Matrix metalloproteinases
AAA: Abdominal aortic aneurysm
rOPN: Recombinant osteopontin
LE: Laminin-type epidermal growth factor-like
HS: Heparan sulphate
CS: Chondroitin sulphate
DS: Dermatan sulphate
HA: Hyaluronic acid
PASMC: Pulmonary artery smooth muscle cells
TNFα: Tumour necrosis factor alpha
IL-1β: Interleukin-1β
LAA: Large artery atherosclerosis
RHAMM: Hyaluronan-mediated motility receptor
HARE: Hyaluronan receptor for endocytosis
LYVE-1: Lymphatic vessel endothelial hyaluronan receptor
HMW-HA: High molecular weight HA
FSAP: Factor VII activating protease
TIMPs: Tissue inhibitors of metalloproteinases
BBB: Blood–brain barrier
LXA4: Lipoxin A4
ROCK: Rho-associated protein kinase
TAZ: Transcriptional co-activator with PDZ-binding motif
Thbs1: Thrombospondin-1
eNOS: Endothelial nitric oxide synthase
MLC: Myosin light chain
IP3: Inositol 1,4,5-trisphosphate
DAG: Diacylglycerol
TGF-β: Transforming growth factor
FGF: Fibroblast growth factor
YAP: Yes-associated protein
MLCK: Myosin light chain kinase
MLCP: Myosin light chain phosphatase
PLC: Phospholipase C
PKC: Protein Kinase C
NO: Nitric oxide
cGMP: Cyclic guanosine monophosphate
PGF2α: Prostaglandin F2α
TXA2: Thromboxane A2
NF-κB: Nuclear factor-kappa B
IL -6: Interleukin-6
IL-8: Interleukin-8
CAFs: Cancer-associated fibroblasts
GTPases: Guanosine triphosphatases
AT1R: Angiotensin II type 1 receptor
ILK: Integrin-linked kinase
LN: Laminin N-terminal globular
MAPK: Mitogen-activated protein kinase
ICAM-1: Intercellular adhesion molecule-1
VCAM-1: Vascular cell adhesion molecule-1
MCP-1: Monocyte chemoattractant protein-1
CAM: Cell adhesion molecule
FAK: Focal adhesion kinase
PI3K: Phosphoinositide 3-kinase
TRAF6: Tumor Necrosis Factor Receptor-Associated Factor 6
NDRG2: N-myc downstream regulated gene 2
PPM1A: Protein phosphatase, Mg2+/Mn2+ dependent 1A
ERK: Extracellular signal-regulated kinases
CCL20: Chemokine (C-C motif) ligand 20
iNOs: Inducible nitric oxide synthase
CXCL1: C-X-C motif chemokine ligand
TREM: Triggering receptor expressed on myeloid cells
ZO-1: Zonula occludens-1
HIF-1α: Hypoxia-inducible factor 1-alpha
AQP-4: Aquaporin-4
H2S: Hydrogen sulfide
TJP: Tight junction protein
MPO: Myeloperoxidase
DDR1: Ddiscoidin domain receptor 1
LDL: Low-density lipoprotein
LRP-1: LDL receptor-related protein-1
